# Supplementary material for: MiniCORVET is a Vps8-containing early endosomal tether in Drosophila
Source: eLife. 2016 Jun 2;5:e14226. doi: 10.7554/eLife.14226 (PMC4935465; doi:10.7554/eLife.14226)
Supplement: Supplementary file 1. — DOI: http://dx.doi.org/10.7554/eLife.14226.024 [file elife-14226-supp1.docx]

1 **ATG**TCGGAGC TTAAGGCCCC GTCGCTGCAA TCTCTGCTGG AATCGGAGCG

Exon: UPPERCASE

Intron: lowercase

51 AGGATCCACA GACAGCCTAC TGGCCGAATC CCTGCAGCTG GACTTCGAAG

101 ATgtgagttg ggattacccg aaaatggaat ggcctagata acactttatc

151 ttgattcatc caattatagC TTGATGATGC CG**AGTTTGCA ATACCGCCCA**

201 CCGATGTGCT GCCTACTTTG GAGGCGGTGC TCAGTGAATT TGAGGCGGAT

gRNA target sequence:
**RED & BOLD**

deleted segment:

**BOLD & UNDERLINE**

251 TCGGATGTGG CCTCCGAGTT TGGAATGCCG GTGCCCCATG CCACGCCCAC

301 GCCCTCGATT GGTGAGGATT CCACGATCAG AACAGACGGC AGAGGTGGTG

351 GAGGATCCAT AATGAGGTAC ACCCTCCTAC ACGGCATCTC GGCACAACTT

401 TCCTCA**GCGG CGGAACGTGT** **CAATGCCGGA G**CAGCCAGTT CCTGTGCCGT

451 GGCCGCATTC ATAGCCATCG GAACATCGCA TGGACACATT TTGAACTTCG

501 ATGTAACCCA AACCCTTAGG TGGGCCCATC AGGACAAGCA TGGTCAGGGT

551 GCGGTGGCCA GTTTGGCTTT CAATGCGGAT TCAACGCGAC TTTTGGCTGG

601 TTTTTCTAGG GGATTGGTTG CCATGCTGGA CACTCATACG GGTGATGTTC

**NEW TRUNCATING STOP CODON** in vps8^1^ due to the 4 bp deletion(pos. 186-189.

651 TCCGTGAGCT GTTCGACGTC ATAACCCCAA ACACCGGCGT ACTGCATGTC

701 AAGTGGACCT CGAGATCATC ACTGGCCTTG TGTGCCGATG CCGGCGGATC

751 GGTGTGGTCT CTTAGTTTCA CCCGGAAGCT GGGCATCCGT GGCTGCCAAT

801 CGAGATGCCT CTTCTCCGGC GCTCGCGGAG AGGTCTGCGC CGTGGAACCT

851 CTAATAATGG ACTCCCAAGG ACGCCATGAA TTGGATCAGT ACTGCATTGT

901 GGCTCTGGCA ACGTTATCCA AGTATTTTAT CGTCACTGTC CGACCAAGAT

951 TAAGGGTCAT CAAATACCAC GTGCTTCAGG GACCTCCTGA TTGCTTACCT

1001 CTCCTCGCCT GGCATTTAGT ACTCATTCAA GCGGCAGATA CTTCCCGCTC

1051 CGTGGACCCC GTGATTGTCG TGGGCCGCGG GAATCAATTA TTCTTTCACC

1101 AACTGTTTGT CTCGAACGGA CGAATAACGC TTTTATACCT TCGACATGTC

1151 CAGCTGCAGG GGAGTCTCCT CTCCGCTCAC TGGCTGGGAC CAAAGTGTGT

1201 TGCCTCATTG GACACGGCGG AAATACTTCA TCTTGTGGAT GTGCGATCCA

1251 GTAAGGAACT AGAATGTATG GATATGGCCA ACGCAGGACT GGTCTACGGA

1301 TCAGCGCAGT TTAAAGGACT AGCCACTGGC GGAAATGTGT CACCTGCCTT

1351 CGCTTTAGCC GGCTCAAATG CCTGTTATAA CTCAGTGGTT TCAAGAGGAA

1401 CCCAGTTGTA CGTTTTAGGC GCCAGGTCCC TGCATATAAT TGGAGTTAGA

1451 ACTTGGTCAG AGAGAATAAG CTTCCTGgta agatcaaaac tgtggattta

1501 aatggaaaat caaagcctca actttatcgt ttctagGTAA AACACCATCG

1551 ATGGCAGGAA GCCTGCCAAC TGGCACTTGA TGGCTACATT GCCTCTGTGG

1601 ATCGCCCGAG GAAACGTGCT CAAGCCAAGG AGCGTATCAT CATGCTTTTT

1651 AAGGAATATA TCGCCAATTC CGCCCGGGCA CCAGAGTATT GTCTCGGCGC

1701 CATTGTTAAC TGCTTGATCA CCGTGGGTGA ACTGGATCTT CTGTGGACAC

1751 AGCTGTGGGA AAAGTTACAC AACAGCAGCA CTGAACTTTT CTTACAGCAC

1801 ATTTCCGAGC ATATAGAAAA GGAAACGATA CATAGTGTTA ATCCAGTGAT

1851 ATCCCAAGCT TTGGTGGATT ACTGGCTGGA ACACTCCCCT GCCAAACTTG

1901 AACAGCTTAT CCTCAAACTG GATTGGATGT GCCTGGATCT CAACCAGGTG

1951 CTTAAGGCTG TGAAGAAGCA TCGTCTGTTT AGGGCTCAAA TCTACCTCAA

2001 CACCCAAGCT TTGAATGACT ATACGGCGGC GCTCACGGAG CTCCTTCCAT

2051 TGGTGACCCC AGATGAGACG GATTTGGGCA ACTGCCTGCT GGTCTACGTT

2101 TCTAGTTGTC TGGCTGGAAG AGAATATCCC AGCGGAGAAA TTCCCGTGGA

2151 GCTGGTGCAT CAAGTCAAGC ACGATGTCCT GCGTTGCCTG ACCTCCCAGC

2201 ATTCCAAGGA GAATGCCGGC GATGAACTGC CCTATCCCTA TCTTAGAGCT

2251 CTCCTCAAGT TCGATACTCG GGAAACCCTA AACGTTATAT CGCTGGCGTT

2301 TCAGGAACGA GAGTTTAGCA ACGAGCTGGG AATCTCGCAT CGCAAGAGGA

2351 TTATCAATCT GCTACTGGAA ATCATGTCGC CCGAGAATGC AACGgtaagg

2401 tttgttaacc ataattgcta gtcatccaac aaaaaactct ttatcttcca

2451 gTGGGCGGAG ATTGGTTGCT TGCTAAACTT CATTGCTCAG CAGATTTCAA

2501 TGCAATGCCT GCCGCGGGAT AGACAGCTTC TGGAACGAGT TTTGAGCCAC

2551 TTGGCGCAGG AGGAAATTGC TAACGAGAGC AGTCGCCAAC ACTCCGAGCG

2601 AGAGAATGCA TGGCACGAGC TACTATCCTC CAACTGCCTG GCCGAGATCA

2651 GCAGCGATGA GGAGCAGCTG CGCTTAGCAG AGAAGGCGAA GTGCTATTGC

2701 GTAGTGGAGT ACCTATTGGA GAAGCTTGAG CGATACGATA CCATCCTGGA

2751 TTCCTACATC CGAAACGAAG CCAGGCACGA AACCATGTTT GCATATATGG

2801 AACGCCATGT TGCTTCACCA AAGAGAAGCA TTTTCCGGCA GTTGAAAAGG

2851 AATCTAAGGG AGCTACTAAC GATCAATGCC AAGGAAACCA CTCGCTTGCT

2901 GTCTCTGCAC TATCCAGAAA AGATTAATGA GCTTTTAGAT AACCTGAGAA

2951 GGGAGGAGAA CTTGTTATAT CTGTTCCTCA AGTGCCTCAA TGATCGTAAA

3001 AGCGAACTGG AAGCCAGTCA AATGGAGTTA CTACTGGAAT TATATTGCAA

3051 AATGGAGTCG TCGTCTACCG TCGAAGAATT CCTTCGATCC AATTCGGGTT

3101 ATCGCTTGGA GAATGCCATT GCCATAGCTG AAAGCCATCA CCTTAACCGA

3151 TCTGTGATTT ATCTGTATGA AAAGCAAGAG AGCTATGCGA AAGCCTTTGA

3201 GTTGTCCATG GAACTGCTTA AGTCCGCTGC TGGCGAGGAA GCAGCCAAGG

3251 AGGCACAGAC TATATCGGCT CTTTTGGCTC GGTCTGTGGA AACGTTACCA

3301 GCCCAGGAAC TGGAACGCTG TTGGTTCGCT CTCTTGCAAT ATATTCTGCC

3351 GCATCAGGAG CTTCAGTCCA TTACCAAATC TCTGCTGCAC GAGGCCTCAC

3401 AGCATATCGA TCTGCATAAT TTGGTTCAAT TGATTATGAA TACCCACAAT

3451 GTGTCAACTA GTTTCGGTGA TATAAAGGAT CTTCTAATGG GTATGCTGGA

3501 CAGTTCGAGG CACAAAACGG AGGCTTTGCG AGCATCTGCT GGAGCCCTGT

3551 GTCAAGATCT TCACCTGAAG TTCGTCAAGC GCTATCAGCA TGCGCATCGT

3601 GGACTTTGGG TGACCACCAC AAAGTGCTCC ATGTGTCGCC AAAGGCTGTA

3651 CGATCACAGC CAGGTGCTAA TATTTGGCGG ATGTGGGCAT GGCATACACG

3701 AGCAGTGCAT GGAGGAGTCG GAGACTCAGT TTGAAGAATG TCCACGGTGC

3751 TTTACGGCCA TTCCAGATCA AAGTATTGGT TTGCCGCGGC CAAATAAAAA

3801 TCTAATCAGC ATTTCCTCGT CCTTGGAAAT GGGTGCCTTG CAACTGAAGG

3851 CACCGCCCAG GCGATTTATA **TAG**
